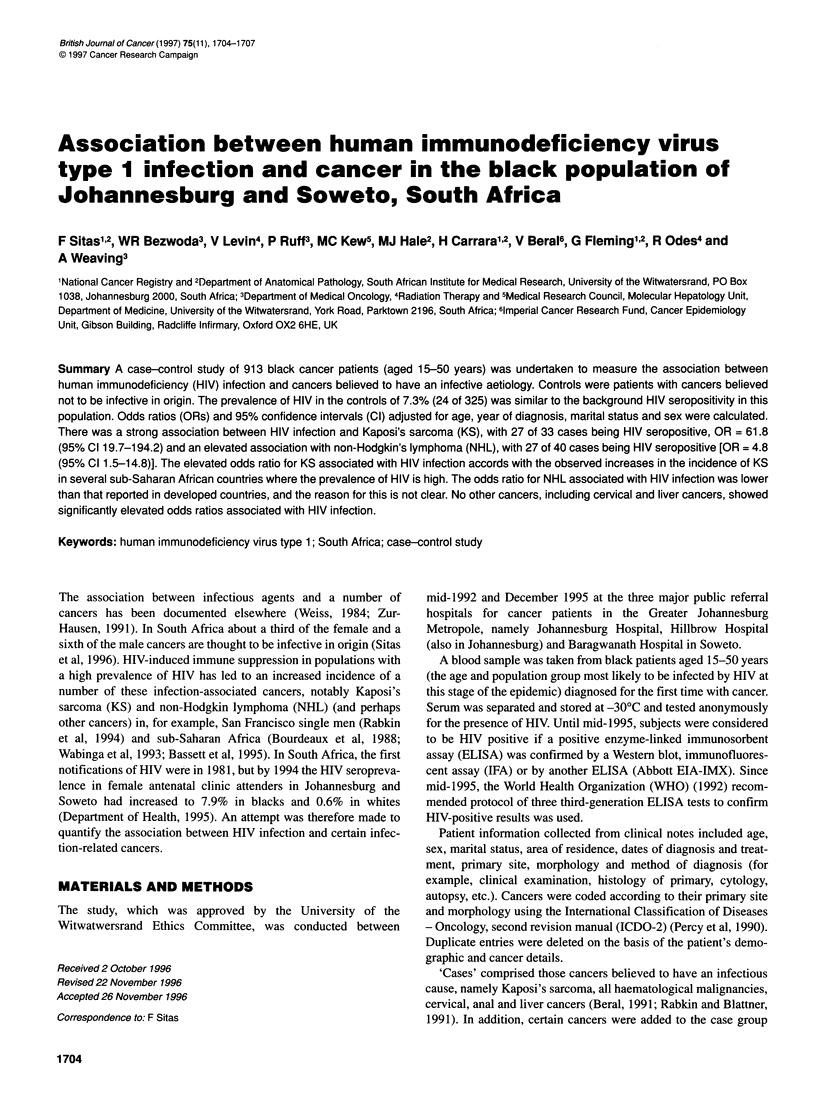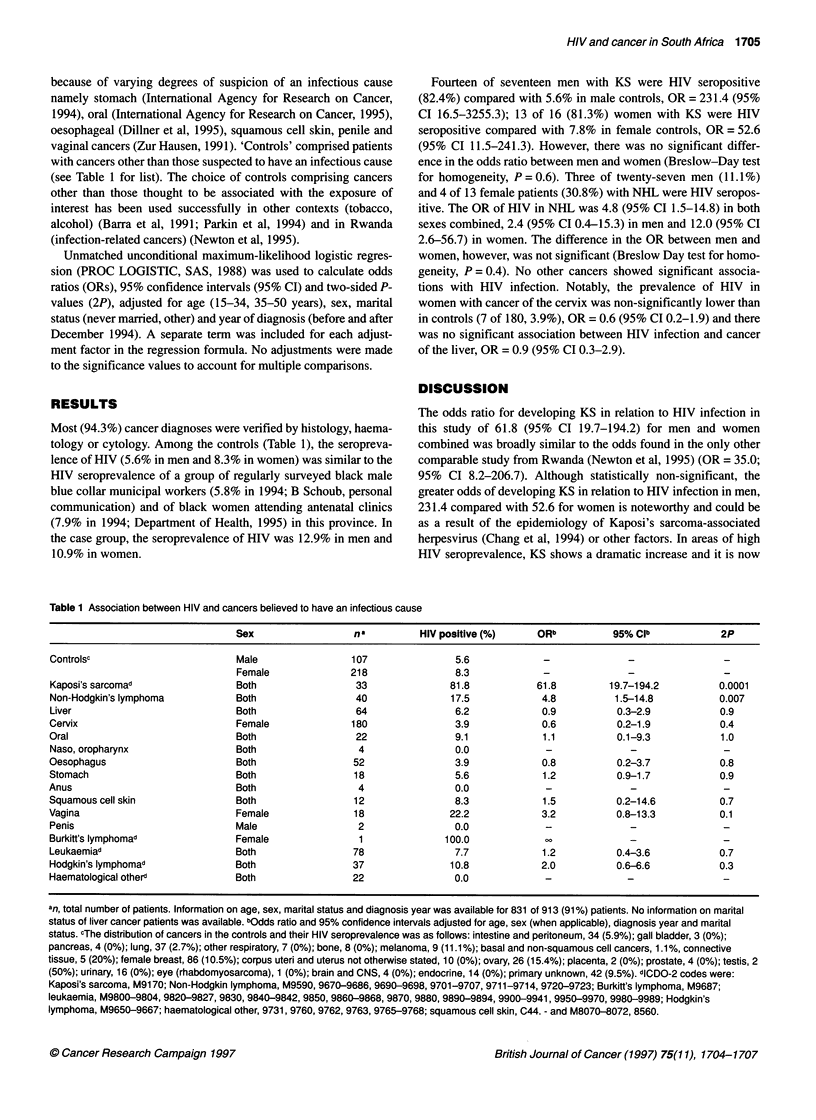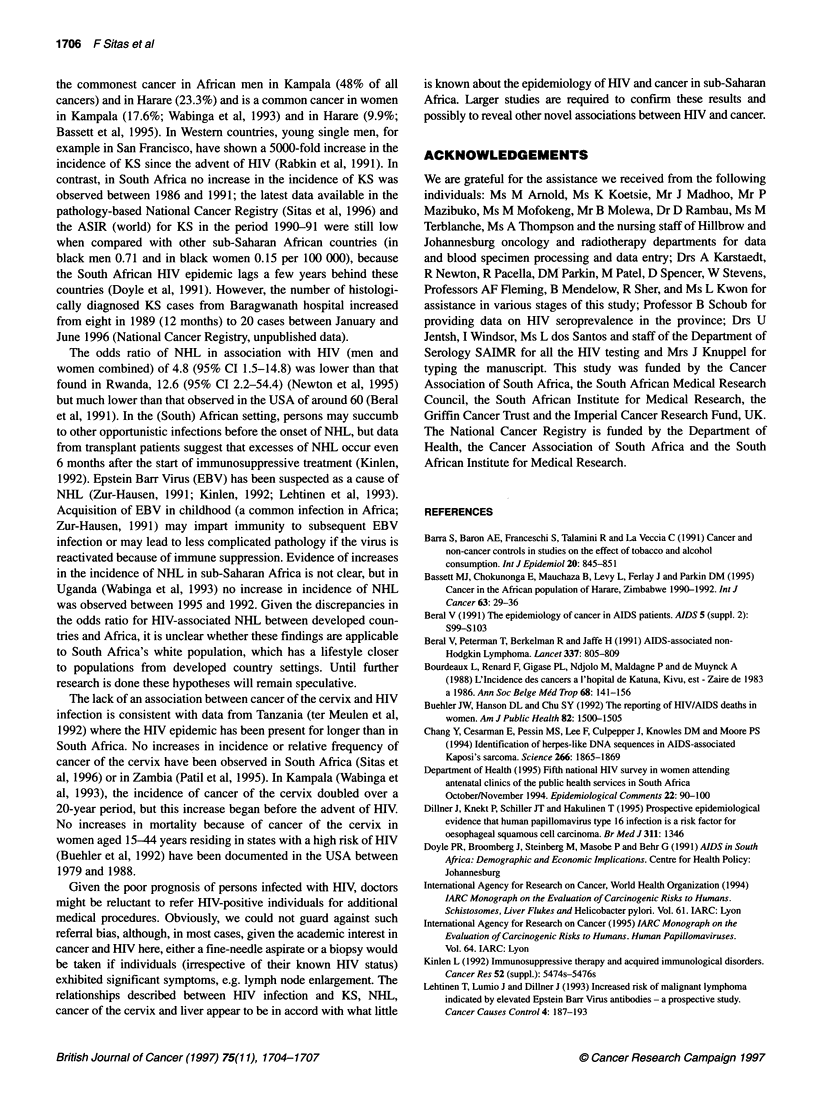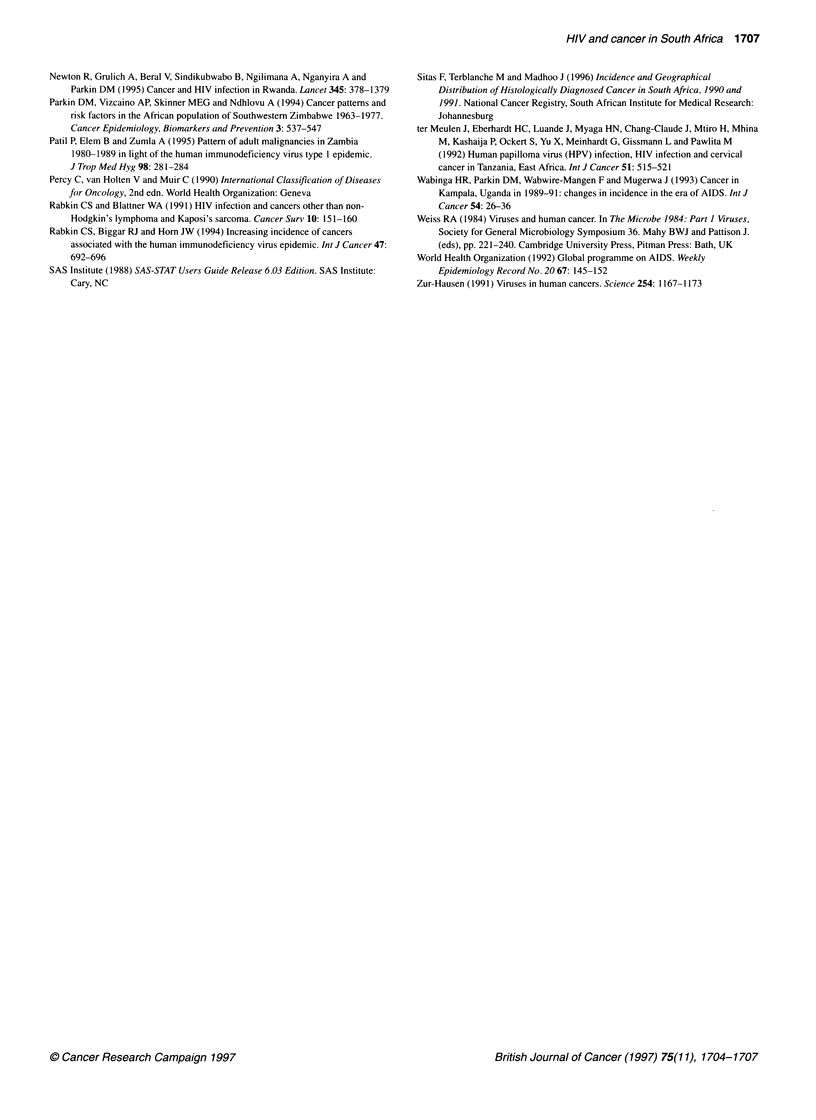# Association between human immunodeficiency virus type 1 infection and cancer in the black population of Johannesburg and Soweto, South Africa.

**DOI:** 10.1038/bjc.1997.290

**Published:** 1997

**Authors:** F. Sitas, W. R. Bezwoda, V. Levin, P. Ruff, M. C. Kew, M. J. Hale, H. Carrara, V. Beral, G. Fleming, R. Odes, A. Weaving

**Affiliations:** National Cancer Registry and Department of Anatomical Pathology, South African Institute for Medical Research, University of the Witwatersrand, Johannesburg.

## Abstract

A case-control study of 913 black cancer patients (aged 15-50 years) was undertaken to measure the association between human immunodeficiency (HIV) infection and cancers believed to have an infective aetiology. Controls were patients with cancers believed not to be infective in origin. The prevalence of HIV in the controls of 7.3% (24 of 325) was similar to the background HIV seropositivity in this population. Odds ratios (ORs) and 95% confidence intervals (CI) adjusted for age, year of diagnosis, marital status and sex were calculated. There was a strong association between HIV infection and Kaposi's sarcoma (KS), with 27 of 33 cases being HIV seropositive, OR = 61.8 (95% CI 19.7-194.2) and an elevated association with non-Hodgkin's lymphoma (NHL), with 27 of 40 cases being HIV seropositive [OR = 4.8 (95% CI 1.5-14.8)]. The elevated odds ratio for KS associated with HIV infection accords with the observed increases in the incidence of KS in several sub-Saharan African countries where the prevalence of HIV is high. The odds ratio for NHL associated with HIV infection was lower than that reported in developed countries, and the reason for this is not clear. No other cancers, including cervical and liver cancers, showed significantly elevated odds ratios associated with HIV infection.


British Journal of Cancer (1997) 75(11), 1704–1707
© 1997 Cancer Research Campaign

### Association between human immunodeficiency virus type 1 infection and cancer in the black population of Johannesburg and Soweto, South Africa

**F Sitas[1,2], WR Bezwoda[3], V Levin[4], P Ruff[3], MC Kew[5], MJ Hale[2], H Carrara[1,2], V Beral[6], G Fleming[1,2], R Odes[4] and A Weaving[3]**

[1]National Cancer Registry and [2]Department of Anatomical Pathology, South African Institute for Medical Research, University of the Witwatersrand, PO Box 1038, Johannesburg 2000, South Africa; [3]Department of Medical Oncology, [4]Radiation Therapy and [5]Medical Research Council, Molecular Hepatology Unit, Department of Medicine, University of the Witwatersrand, York Road, Parktown 2196, South Africa; [6]Imperial Cancer Research Fund, Cancer Epidemiology Unit, Gibson Building, Radcliffe Infirmary, Oxford OX2 6HE, UK

**Summary** A case–control study of 913 black cancer patients (aged 15–50 years) was undertaken to measure the association between human immunodeficiency (HIV) infection and cancers believed to have an infective aetiology. Controls were patients with cancers believed not to be infective in origin. The prevalence of HIV in the controls of 7.3% (24 of 325) was similar to the background HIV seropositivity in this population. Odds ratios (ORs) and 95% confidence intervals (CI) adjusted for age, year of diagnosis, marital status and sex were calculated. There was a strong association between HIV infection and Kaposi's sarcoma (KS), with 27 of 33 cases being HIV seropositive, OR = 61.8 (95% CI 19.7–194.2) and an elevated association with non-Hodgkin's lymphoma (NHL), with 27 of 40 cases being HIV seropositive [OR = 4.8 (95% CI 1.5–14.8)]. The elevated odds ratio for KS associated with HIV infection accords with the observed increases in the incidence of KS in several sub-Saharan African countries where the prevalence of HIV is high. The odds ratio for NHL associated with HIV infection was lower than that reported in developed countries, and the reason for this is not clear. No other cancers, including cervical and liver cancers, showed significantly elevated odds ratios associated with HIV infection.

**Keywords:** human immunodeficiency virus type 1; South Africa; case–control study

The association between infectious agents and a number of cancers has been documented elsewhere (Weiss, 1984; Zur-Hausen, 1991). In South Africa about a third of the female and a sixth of the male cancers are thought to be infective in origin (Sitas et al, 1996). HIV-induced immune suppression in populations with a high prevalence of HIV has led to an increased incidence of a number of these infection-associated cancers, notably Kaposi's sarcoma (KS) and non-Hodgkin lymphoma (NHL) (and perhaps other cancers) in, for example, San Francisco single men (Rabkin et al, 1994) and sub-Saharan Africa (Bourdeaux et al, 1988; Wabinga et al, 1993; Bassett et al, 1995). In South Africa, the first notifications of HIV were in 1981, but by 1994 the HIV seroprevalence in female antenatal clinic attenders in Johannesburg and Soweto had increased to 7.9% in blacks and 0.6% in whites (Department of Health, 1995). An attempt was therefore made to quantify the association between HIV infection and certain infection-related cancers.

### MATERIALS AND METHODS

The study, which was approved by the University of the Witwatwersrand Ethics Committee, was conducted between mid-1992 and December 1995 at the three major public referral hospitals for cancer patients in the Greater Johannesburg Metropole, namely Johannesburg Hospital, Hillbrow Hospital (also in Johannesburg) and Baragwanath Hospital in Soweto.

A blood sample was taken from black patients aged 15–50 years (the age and population group most likely to be infected by HIV at this stage of the epidemic) diagnosed for the first time with cancer. Serum was separated and stored at –30°C and tested anonymously for the presence of HIV. Until mid-1995, subjects were considered to be HIV positive if a positive enzyme-linked immunosorbent assay (ELISA) was confirmed by a Western blot, immunofluorescent assay (IFA) or by another ELISA (Abbott EIA-IMX). Since mid-1995, the World Health Organization (WHO) (1992) recommended protocol of three third-generation ELISA tests to confirm HIV-positive results was used.

Patient information collected from clinical notes included age, sex, marital status, area of residence, dates of diagnosis and treatment, primary site, morphology and method of diagnosis (for example, clinical examination, histology of primary, cytology, autopsy, etc.). Cancers were coded according to their primary site and morphology using the International Classification of Diseases – Oncology, second revision manual (ICDO-2) (Percy et al, 1990). Duplicate entries were deleted on the basis of the patient's demographic and cancer details.

'Cases' comprised those cancers believed to have an infectious cause, namely Kaposi's sarcoma, all haematological malignancies, cervical, anal and liver cancers (Beral, 1991; Rabkin and Blattner, 1991). In addition, certain cancers were added to the case group

*Received 2 October 1996*
*Revised 22 November 1996*
*Accepted 26 November 1996*

*Correspondence to:* F Sitas because of varying degrees of suspicion of an infectious cause namely stomach (International Agency for Research on Cancer, 1994), oral (International Agency for Research on Cancer, 1995), oesophageal (Dillner et al, 1995), squamous cell skin, penile and vaginal cancers (Zur Hausen, 1991). 'Controls' comprised patients with cancers other than those suspected to have an infectious cause (see Table 1 for list). The choice of controls comprising cancers other than those thought to be associated with the exposure of interest has been used successfully in other contexts (tobacco, alcohol) (Barra et al, 1991; Parkin et al, 1994) and in Rwanda (infection-related cancers) (Newton et al, 1995).

Unmatched unconditional maximum-likelihood logistic regression (PROC LOGISTIC, SAS, 1988) was used to calculate odds ratios (ORs), 95% confidence intervals (95% CI) and two-sided *P*-values (2*P*), adjusted for age (15–34, 35–50 years), sex, marital status (never married, other) and year of diagnosis (before and after December 1994). A separate term was included for each adjustment factor in the regression formula. No adjustments were made to the significance values to account for multiple comparisons.

### RESULTS

Most (94.3%) cancer diagnoses were verified by histology, haematology or cytology. Among the controls (Table 1), the seroprevalence of HIV (5.6% in men and 8.3% in women) was similar to the HIV seroprevalence of a group of regularly surveyed black male blue collar municipal workers (5.8% in 1994; B Schoub, personal communication) and of black women attending antenatal clinics (7.9% in 1994; Department of Health, 1995) in this province. In the case group, the seroprevalence of HIV was 12.9% in men and 10.9% in women.

Fourteen of seventeen men with KS were HIV seropositive (82.4%) compared with 5.6% in male controls, OR = 231.4 (95% CI 16.5–3255.3); 13 of 16 (81.3%) women with KS were HIV seropositive compared with 7.8% in female controls, OR = 52.6 (95% CI 11.5–241.3). However, there was no significant difference in the odds ratio between men and women (Breslow–Day test for homogeneity, *P* = 0.6). Three of twenty-seven men (11.1%) and 4 of 13 female patients (30.8%) with NHL were HIV seropositive. The OR of HIV in NHL was 4.8 (95% CI 1.5–14.8) in both sexes combined, 2.4 (95% CI 0.4–15.3) in men and 12.0 (95% CI 2.6–56.7) in women. The difference in the OR between men and women, however, was not significant (Breslow Day test for homogeneity, *P* = 0.4). No other cancers showed significant associations with HIV infection. Notably, the prevalence of HIV in women with cancer of the cervix was non-significantly lower than in controls (7 of 180, 3.9%), OR = 0.6 (95% CI 0.2–1.9) and there was no significant association between HIV infection and cancer of the liver, OR = 0.9 (95% CI 0.3–2.9).

### DISCUSSION

The odds ratio for developing KS in relation to HIV infection in this study of 61.8 (95% CI 19.7–194.2) for men and women combined was broadly similar to the odds found in the only other comparable study from Rwanda (Newton et al, 1995) (OR = 35.0; 95% CI 8.2–206.7). Although statistically non-significant, the greater odds of developing KS in relation to HIV infection in men, 231.4 compared with 52.6 for women is noteworthy and could be as a result of the epidemiology of Kaposi's sarcoma-associated herpesvirus (Chang et al, 1994) or other factors. In areas of high HIV seroprevalence, KS shows a dramatic increase and it is now

**Table 1** Association between HIV and cancers believed to have an infectious cause

| | Sex | *n*[a] | HIV positive (%) | OR[b] | 95% CI[b] | 2*P* |
|---|---|---|---|---|---|---|
| Controls[c] | Male | 107 | 5.6 | – | – | – |
| | Female | 218 | 8.3 | – | – | – |
| Kaposi's sarcoma[d] | Both | 33 | 81.8 | 61.8 | 19.7–194.2 | 0.0001 |
| Non-Hodgkin's lymphoma | Both | 40 | 17.5 | 4.8 | 1.5–14.8 | 0.007 |
| Liver | Both | 64 | 6.2 | 0.9 | 0.3–2.9 | 0.9 |
| Cervix | Female | 180 | 3.9 | 0.6 | 0.2–1.9 | 0.4 |
| Oral | Both | 22 | 9.1 | 1.1 | 0.1–9.3 | 1.0 |
| Naso, oropharynx | Both | 4 | 0.0 | – | – | – |
| Oesophagus | Both | 52 | 3.9 | 0.8 | 0.2–3.7 | 0.8 |
| Stomach | Both | 18 | 5.6 | 1.2 | 0.9–1.7 | 0.9 |
| Anus | Both | 4 | 0.0 | – | – | – |
| Squamous cell skin | Both | 12 | 8.3 | 1.5 | 0.2–14.6 | 0.7 |
| Vagina | Female | 18 | 22.2 | 3.2 | 0.8–13.3 | 0.1 |
| Penis | Male | 2 | 0.0 | – | – | – |
| Burkitt's lymphoma[d] | Female | 1 | 100.0 | ∞ | – | – |
| Leukaemia[d] | Both | 78 | 7.7 | 1.2 | 0.4–3.6 | 0.7 |
| Hodgkin's lymphoma[d] | Both | 37 | 10.8 | 2.0 | 0.6–6.6 | 0.3 |
| Haematological other[d] | Both | 22 | 0.0 | – | – | – |

[a]*n*, total number of patients. Information on age, sex, marital status and diagnosis year was available for 831 of 913 (91%) patients. No information on marital status of liver cancer patients was available. [b]Odds ratio and 95% confidence intervals adjusted for age, sex (when applicable), diagnosis year and marital status. [c]The distribution of cancers in the controls and their HIV seroprevalence was as follows: intestine and peritoneum, 34 (5.9%); gall bladder, 3 (0%); pancreas, 4 (0%); lung, 37 (2.7%); other respiratory, 7 (0%); bone, 8 (0%); melanoma, 9 (11.1%); basal and non-squamous cell cancers, 1.1%, connective tissue, 5 (20%); female breast, 86 (10.5%); corpus uteri and uterus not otherwise stated, 10 (0%); ovary, 26 (15.4%); placenta, 2 (0%); prostate, 4 (0%); testis, 2 (50%); urinary, 16 (0%); eye (rhabdomyosarcoma), 1 (0%); brain and CNS, 4 (0%); endocrine, 14 (0%); primary unknown, 42 (9.5%). [d]ICDO-2 codes were: Kaposi's sarcoma, M9170; Non-Hodgkin lymphoma, M9590, 9670–9686, 9690–9698, 9701–9707, 9711–9714, 9720–9723; Burkitt's lymphoma, M9687; leukaemia, M9800–9804, 9820–9827, 9830, 9840–9842, 9850, 9860–9868, 9870, 9880, 9890–9894, 9900–9941, 9950–9970, 9980–9989; Hodgkin's lymphoma, M9650–9667; haematological other, 9731, 9760, 9762, 9763, 9765–9768; squamous cell skin, C44. - and M8070–8072, 8560.

© Cancer Research Campaign 1997

the commonest cancer in African men in Kampala (48% of all cancers) and in Harare (23.3%) and is a common cancer in women in Kampala (17.6%; Wabinga et al, 1993) and in Harare (9.9%; Bassett et al, 1995). In Western countries, young single men, for example in San Francisco, have shown a 5000-fold increase in the incidence of KS since the advent of HIV (Rabkin et al, 1991). In contrast, in South Africa no increase in the incidence of KS was observed between 1986 and 1991; the latest data available in the pathology-based National Cancer Registry (Sitas et al, 1996) and the ASIR (world) for KS in the period 1990–91 were still low when compared with other sub-Saharan African countries (in black men 0.71 and in black women 0.15 per 100 000), because the South African HIV epidemic lags a few years behind these countries (Doyle et al, 1991). However, the number of histologically diagnosed KS cases from Baragwanath hospital increased from eight in 1989 (12 months) to 20 cases between January and June 1996 (National Cancer Registry, unpublished data).

The odds ratio of NHL in association with HIV (men and women combined) of 4.8 (95% CI 1.5–14.8) was lower than that found in Rwanda, 12.6 (95% CI 2.2–54.4) (Newton et al, 1995) but much lower than that observed in the USA of around 60 (Beral et al, 1991). In the (South) African setting, persons may succumb to other opportunistic infections before the onset of NHL, but data from transplant patients suggest that excesses of NHL occur even 6 months after the start of immunosuppressive treatment (Kinlen, 1992). Epstein Barr Virus (EBV) has been suspected as a cause of NHL (Zur-Hausen, 1991; Kinlen, 1992; Lehtinen et al, 1993). Acquisition of EBV in childhood (a common infection in Africa; Zur-Hausen, 1991) may impart immunity to subsequent EBV infection or may lead to less complicated pathology if the virus is reactivated because of immune suppression. Evidence of increases in the incidence of NHL in sub-Saharan Africa is not clear, but in Uganda (Wabinga et al, 1993) no increase in incidence of NHL was observed between 1995 and 1992. Given the discrepancies in the odds ratio for HIV-associated NHL between developed countries and Africa, it is unclear whether these findings are applicable to South Africa's white population, which has a lifestyle closer to populations from developed country settings. Until further research is done these hypotheses will remain speculative.

The lack of an association between cancer of the cervix and HIV infection is consistent with data from Tanzania (ter Meulen et al, 1992) where the HIV epidemic has been present for longer than in South Africa. No increases in incidence or relative frequency of cancer of the cervix have been observed in South Africa (Sitas et al, 1996) or in Zambia (Patil et al, 1995). In Kampala (Wabinga et al, 1993), the incidence of cancer of the cervix doubled over a 20-year period, but this increase began before the advent of HIV. No increases in mortality because of cancer of the cervix in women aged 15–44 years residing in states with a high risk of HIV (Buehler et al, 1992) have been documented in the USA between 1979 and 1988.

Given the poor prognosis of persons infected with HIV, doctors might be reluctant to refer HIV-positive individuals for additional medical procedures. Obviously, we could not guard against such referral bias, although, in most cases, given the academic interest in cancer and HIV here, either a fine-needle aspirate or a biopsy would be taken if individuals (irrespective of their known HIV status) exhibited significant symptoms, e.g. lymph node enlargement. The relationships described between HIV infection and KS, NHL, cancer of the cervix and liver appear to be in accord with what little is known about the epidemiology of HIV and cancer in sub-Saharan Africa. Larger studies are required to confirm these results and possibly to reveal other novel associations between HIV and cancer.

### ACKNOWLEDGEMENTS

We are grateful for the assistance we received from the following individuals: Ms M Arnold, Ms K Koetsie, Mr J Madhoo, Mr P Mazibuko, Ms M Mofokeng, Mr B Molewa, Dr D Rambau, Ms M Terblanche, Ms A Thompson and the nursing staff of Hillbrow and Johannesburg oncology and radiotherapy departments for data and blood specimen processing and data entry; Drs A Karstaedt, R Newton, R Pacella, DM Parkin, M Patel, D Spencer, W Stevens, Professors AF Fleming, B Mendelow, R Sher, and Ms L Kwon for assistance in various stages of this study; Professor B Schoub for providing data on HIV seroprevalence in the province; Drs U Jentsh, I Windsor, Ms L dos Santos and staff of the Department of Serology SAIMR for all the HIV testing and Mrs J Knuppel for typing the manuscript. This study was funded by the Cancer Association of South Africa, the South African Medical Research Council, the South African Institute for Medical Research, the Griffin Cancer Trust and the Imperial Cancer Research Fund, UK. The National Cancer Registry is funded by the Department of Health, the Cancer Association of South Africa and the South African Institute for Medical Research.

### REFERENCES

Barra S, Baron AE, Franceschi S, Talamini R and La Veccia C (1991) Cancer and non-cancer controls in studies on the effect of tobacco and alcohol consumption. *Int J Epidemiol* **20**: 845–851

Bassett MJ, Chokunonga E, Mauchaza B, Levy L, Ferlay J and Parkin DM (1995) Cancer in the African population of Harare, Zimbabwe 1990–1992. *Int J Cancer* **63**: 29–36

Beral V (1991) The epidemiology of cancer in AIDS patients. *AIDS* **5** (suppl. 2): S99–S103

Beral V, Peterman T, Berkelman R and Jaffe H (1991) AIDS-associated non-Hodgkin Lymphoma. *Lancet* **337**: 805–809

Bourdeaux L, Renard F, Gigase PL, Ndjolo M, Maldagne P and de Muynck A (1988) L'Incidence des cancers a l'hopital de Katuna, Kivu, est - Zaire de 1983 a 1986. *Ann Soc Belge Méd Trop* **68**: 141–156

Buehler JW, Hanson DL and Chu SY (1992) The reporting of HIV/AIDS deaths in women. *Am J Public Health* **82**: 1500–1505

Chang Y, Cesarman E, Pessin MS, Lee F, Culpepper J, Knowles DM and Moore PS (1994) Identification of herpes-like DNA sequences in AIDS-associated Kaposi's sarcoma. *Science* **266**: 1865–1869

Department of Health (1995) Fifth national HIV survey in women attending antenatal clinics of the public health services in South Africa October/November 1994. *Epidemiological Comments* **22**: 90–100

Dillner J, Knekt P, Schiller JT and Hakulinen T (1995) Prospective epidemiological evidence that human papillomavirus type 16 infection is a risk factor for oesophageal squamous cell carcinoma. *Br Med J* **311**: 1346

Doyle PR, Broomberg J, Steinberg M, Masobe P and Behr G (1991) *AIDS in South Africa: Demographic and Economic Implications*. Centre for Health Policy: Johannesburg

International Agency for Research on Cancer, World Health Organization (1994) *IARC Monograph on the Evaluation of Carcinogenic Risks to Humans. Schistosomes, Liver Flukes and* Helicobacter pylori. Vol. 61. IARC: Lyon

International Agency for Research on Cancer (1995) *IARC Monograph on the Evaluation of Carcinogenic Risks to Humans. Human Papillomaviruses.* Vol. 64. IARC: Lyon

Kinlen L (1992) Immunosuppressive therapy and acquired immunological disorders. *Cancer Res* **52** (suppl.): 5474s–5476s

Lehtinen T, Lumio J and Dillner J (1993) Increased risk of malignant lymphoma indicated by elevated Epstein Barr Virus antibodies – a prospective study. *Cancer Causes Control* **4**: 187–193

© Cancer Research Campaign 1997

Newton R, Grulich A, Beral V, Sindikubwabo B, Ngilimana A, Nganyira A and Parkin DM (1995) Cancer and HIV infection in Rwanda. *Lancet* **345**: 378–1379

Parkin DM, Vizcaino AP, Skinner MEG and Ndhlovu A (1994) Cancer patterns and risk factors in the African population of Southwestern Zimbabwe 1963–1977. *Cancer Epidemiology, Biomarkers and Prevention* **3**: 537–547

Patil P, Elem B and Zumla A (1995) Pattern of adult malignancies in Zambia 1980–1989 in light of the human immunodeficiency virus type 1 epidemic. *J Trop Med Hyg* **98**: 281–284

Percy C, van Holten V and Muir C (1990) *International Classification of Diseases for Oncology*, 2nd edn. World Health Organization: Geneva

Rabkin CS and Blattner WA (1991) HIV infection and cancers other than non-Hodgkin's lymphoma and Kaposi's sarcoma. *Cancer Surv* **10**: 151–160

Rabkin CS, Biggar RJ and Horn JW (1994) Increasing incidence of cancers associated with the human immunodeficiency virus epidemic. *Int J Cancer* **47**: 692–696

SAS Institute (1988) *SAS-STAT Users Guide Release 6.03 Edition*. SAS Institute: Cary, NC

Sitas F, Terblanche M and Madhoo J (1996) *Incidence and Geographical Distribution of Histologically Diagnosed Cancer in South Africa, 1990 and 1991*. National Cancer Registry, South African Institute for Medical Research: Johannesburg ter Meulen J, Eberhardt HC, Luande J, Myaga HN, Chang-Claude J, Mtiro H, Mhina M, Kashaija P, Ockert S, Yu X, Meinhardt G, Gissmann L and Pawlita M (1992) Human papilloma virus (HPV) infection, HIV infection and cervical cancer in Tanzania, East Africa. *Int J Cancer* **51**: 515–521

Wabinga HR, Parkin DM, Wabwire-Mangen F and Mugerwa J (1993) Cancer in Kampala, Uganda in 1989–91: changes in incidence in the era of AIDS. *Int J Cancer* **54**: 26–36

Weiss RA (1984) Viruses and human cancer. In *The Microbe 1984: Part 1 Viruses*, Society for General Microbiology Symposium 36. Mahy BWJ and Pattison J. (eds), pp. 221–240. Cambridge University Press, Pitman Press: Bath, UK

World Health Organization (1992) Global programme on AIDS. *Weekly Epidemiology Record No. 20* **67**: 145–152

Zur-Hausen (1991) Viruses in human cancers. *Science* **254**: 1167–1173

© Cancer Research Campaign 1997